# Editorial: *Spiroplasma, Mycoplasma*, Phytoplasma, and other genome-reduced and wall-less mollicutes: their genetics, genomics, mechanics, interactions and symbiosis with insects, other animals and plants

**DOI:** 10.3389/fmicb.2024.1477536

**Published:** 2024-08-30

**Authors:** Takema Fukatsu, Shigeyuki Kakizawa, Toshiyuki Harumoto, Akiko Sugio, Chih-Horng Kuo

**Affiliations:** ^1^Bioproduction Research Institute, National Institute of Advanced Industrial Science and Technology, Tsukuba, Japan; ^2^Graduate School of Life and Environmental Sciences, University of Tsukuba, Tsukuba, Japan; ^3^Department of Biological Sciences, Graduate School of Science, The University of Tokyo, Tokyo, Japan; ^4^Hakubi Center for Advanced Research, Kyoto University, Kyoto, Japan; ^5^Graduate School of Biostudies, Kyoto University, Kyoto, Japan; ^6^IGEPP, INRAE, Institut Agro, University of Rennes, Le Rheu, France; ^7^Institute of Plant and Microbial Biology, Academia Sinica, Taipei, Taiwan

**Keywords:** *Spiroplasma*, *Mycoplasma*, Phytoplasma, symbiont, pathogen, genome, insect, plant

Genome-reduced, wall-less, and fastidious bacteria of the genera *Spiroplasma, Mycoplasma*, “*Candidatus* Phytoplasma” and allies belonging to the class Mollicutes, are known for a number of unique microbiological features, which have prompted researchers to investigate their basic, applied, and medical aspects (Brown et al., 2018). They are mostly parasitic or symbiotic to a variety of animals or plants, living on or within the eukaryotic cells. Spiroplasmas, recognized by their characteristic helical shapes and active twitching motility, are associated with diverse arthropods and plants (Gasparich et al., 2020), and have been developed as models for the study of facultative symbionts (Anbutsu and Fukatsu, 2011; Lo et al., 2016). Some strains of *Spiroplasma poulsonii* and *Spiroplasma ixodetis* cause a remarkable reproductive phenotype, called male-killing, of their insect hosts (Hurst and Frost, 2015). In contrast, some other insect-associated spiroplasmas protect their hosts from natural enemies, including parasitoid wasps, nematodes, and pathogenic fungi (Ballinger and Perlman, 2019). *Spiroplasma citri* and *Spiroplasma kunkelii* are notorious as devastating pathogens of citrus and maize, respectively (Gasparich et al., 2020). Mycoplasmas are not only medically important as human or animal pathogens like *Mycoplasma pneumoniae* (Waites and Talkington, 2004) and *Mycoplasma mycoides* (Teodoro et al., 2020), but also intensively investigated as minimal-genome bacterial models (Yus et al., 2009). Microbial genome synthesis and engineering technologies have been developed mainly on *M. mycoides* and *Mycoplasma capricolum* (Venter et al., 2022). Some mycoplasmas are known for their capability of unique gliding motility (Miyata et al., 2020). Phytoplasmas are obligatorily parasitic to plant phloem tissues and vectored by plant-sucking insects, often causing spectacular plant morphological changes like phyllody, virescence, witches' bloom, etc. (Hogenhout et al., 2008; Bertaccini et al., 2022).

While the conventional studies have revealed fascinating aspects of this bizarre bacterial group, the whole picture of their diversity and versatility has long been elusive mainly due to their reluctance to axenic cultivation. However, owing to the recent development and availability of high-throughput DNA sequencing technologies, our knowledge on the diversity of such fastidious microbes in a variety of environments has been growing rapidly. In this context, the Research Topic “*Spiroplasma, Mycoplasma, Phytoplasma, and other genome-reduced and wall-less mollicutes: their genetics, genomics, mechanics, interactions and symbiosis with insects, other animals and plants*” is aimed to provide an opportunity to compile the new information emerging in this research field. In total, 10 articles and one mini review were published, of which five, three, and three articles are on *Spiroplasma, Mycoplasma*, Phytoplasma, respectively, thereby covering this research area in a balanced manner.

As for *Spiroplasma*, unique articles are contributed to this Research Topic. Mizutani, Omori et al. reported successful cloning of the whole 1.12 Mbp genome of *Spiroplasma chrysopicola*, which was originally isolated from a deer fly *Chrysops* sp., into the yeast *Saccharomyces cerevisiae*. Now the *S. chrysopicola* genome is retained in yeast cells, can be genetically engineered using sophisticated genetic tools available for *S. cerevisiae*, and can be distributed to anybody and utilized for further research. While a series of elaborate synthetic biological technologies have been developed using *Mycoplasma* spp. (Venter et al., 2022), this study serves as an initial step toward the synthetic biological approaches to *Spiroplasma* spp. Following two articles reported the characterization of newly obtained genomes of *S. ixodetis* strains. Arai et al. analyzed the genome sequence of a male-killing *S. ixodetis* symbiont of *Homona magnanima*. The genome harbored a number of putative virulence-associated genes like ankyrin domain containing genes, while no homologous sequence of *spaid*, a male-killing gene of *S. poulsonii* in *Drosophila* (Harumoto and Lemaitre, 2018), was identified. This implicates the diverged mechanism of male killing evolved in distinct *Spiroplasma* symbionts. The authors also showed bacterial tropism toward host somatic tissues and successful proliferation in various inset cell lines, which conform to the fact that *S*. *ixodetis* strains have a broad host range. Moore and Ballinger presented the complete genome sequence of a defensive *S. ixodetis* symbiont of *D. atripex* and revealed a set of toxins and virulence genes containing ribosome-inactivating protein toxin (RIP), OTU-like cysteine protease, ankyrin, and other bacterial toxin domains. They also performed a genus-wide comparative analysis of toxin/virulence-related domains between vertically transmitted and non-vertically transmitted strains, then identified a conserved core of toxin domains that is specific to the vertically transmitted strains. Kakizawa et al. highlighted the diversity of *Spiroplasma* as a group of facultative symbiotic bacteria of insects. The authors surveyed diverse stinkbugs representing 13 families, 69 genera, 97 species and 468 individuals, and detected *Spiroplasma* infection from four families (30.8%), seven genera (10.1%), 11 species (11.3%) and 21 individuals (4.5%). Phylogenetically, the stinkbug-associated *Spiroplasma* symbionts were placed in three distinct clades in the Spiroplasmataceae, confirming multiple and dynamic evolutionary trajectories of the stinkbug-*Spiroplasma* associations.

As for *Mycoplasma*, Mizutani, Sasajima et al. contributed a sophisticated biophysical work on the bacterial unique motility. *Mycoplasma pneumoniae*, known as the causative agent of mycoplasma pneumonia, binds to sialylated oligosaccharides and glides on host cell surface, which is essential for initiating the infection process (Miyata and Hamaguchi, 2016). The authors measured the stall force and the gliding speed of each *M. pneumoniae* cell carrying a bead that was manipulatable using optical tweezers. From the measurements of the faster strain M129 and the slower strain FH, detailed parameters of the gliding motility were measured. These results provide fundamental parameters underlying the bacterial gliding movement. Ras et al. conducted comparative genomic survey of *Mycoplasma* spp. for biosynthesis pathway genes of coenzyme A. The authors showed that most *Mycoplasma* genomes retain the genetic capacity to synthesize coenzyme A, but there was a differentiated prevalence of these genes across species. The final enzyme gene in the biosynthesis pathway encoding dephospho-coenzyme A kinase was found to be the most common among the studied *Mycoplasma* genomes. Yang et al. contributed a mini review about a comprehensive summary of lncRNAs and the responses of host cells associated with *M. pneumoniae* infections. With increasing literature on this topic, the review provided insights into the protective roles of lncRNAs against various forms of *M. pneumoniae* infections. They also discussed the involvement of lncRNAs in cardiovascular diseases, neurological disorders, cancers, and diabetes. Despite identifying key lncRNAs linked to *M. pneumoniae* pneumonia, their biological roles and mechanisms remain largely unknown. The authors emphasize the importance of understanding the molecular mechanisms of these lncRNAs.

As for Phytoplasma, two research articles provided technological advancements to facilitate the whole genome sequencing of these uncultivated pathogens from the infected plant samples. Tan et al. described an immunoprecipitation-based method for enriching the phytoplasma cells prior to DNA extraction. Zhang et al. described another enrichment method for phytoplasma cells based on serial filtration, as well as removal of host DNA using DNase I prior to the lysis of bacterial cells. By combining short read sequencing based on the Illumina platform and long read sequencing based on the Oxford Nanopore Technologies platform, both methods are effective for obtaining complete assemblies of these repeat-rich and compositionally biased genomes from metagenomic sequencing. It is worth noting that another enrichment protocol using iodixanol density gradients was developed recently and also shown to be effective (Jardim et al., 2022). In the third phytoplasma research article in this Research Topic, Huang et al. reported the first complete genome sequence of “*Candidatus* Phytoplasma luffae” and found that a pair of 75 kb repeats and at least 13 potential mobile units (PMUs) account for ~25% of this 769 kb chromosome. PMUs are phytoplasma-specific mobile genetic elements that often associate with effector genes (Bai et al., 2006, 2009) and likely contribute to the horizontal transfer of these virulence factors (Chung et al., 2013). In this new work, a genus-wide analysis of PMUs established a classification scheme of these mobile elements and identified strong correlations between PMU abundance and genome size at both within- and between-species levels.

In conclusion, the Research Topic presents a valuable overview of the current research coverage on *Spiroplasma, Mycoplasma*, and Phytoplasma, which encompasses such diverse areas as microbial diversity, genomics, reproductive manipulation, defense, toxins, biophysics and synthetic biology. These contributions highlight a variety of interesting biological phenomena observed with this wall-less, fastidious, and host-associated bacterial group. We hope that this Research Topic would provide some insight into what directions are promising in our future studies.
